# Progress of Cholera

**Published:** 1849-07

**Authors:** 


### PROGRESS OF CHOLERA.

As was generally apprehended, cholera, since the commencement of hot weather, has made great progress in the Valley of the Mississippi. In New Orleans we no longer hear of the epidemic. It may be hoped that it has spent its force upon that ill-fated city, and will not soon return to it. Along the Mississippi, on the plantations, the ravages of the disease among the negroes is represented as having been frightful.

In St. Louis, the mortality has far exceeded that experienced from the epidemic in New Orleans. On the 21st of June the number of deaths had reached a hundred a day, and in a week from 700 to 1000 are reported to have died. Among the victims were some of the best citizens—the disease not being confined to the indigent and dissipated, nor to foreigners and those crowded into damp and filthy situations, although it cannot be doubted that the mortality was greatly increased by the immense number of immigrants pressing into St. Louis. The thermometer, in the shade, stood at 90° during the days when the pestilence was at its height.

About the middle of the month the epidemic reappeared at Nashville in a malignant form, and the mortality for a few days was alarming; but after a short reign it appears to have again subsided. Near the close of the month the number of fatal cases did not average more than three a day. It is worthy of special notice in reference to this city, that the disease was confined to a limited district. Nearly all the malignant cases occurred on the hill south of the University. In that neighborhood reside many of the best families in Nashville; and there died Prof. Hamilton and three sisters in the same house. While such was the fatality at that spot, the city proper was nearly exempt from cholera.

In Cincinnati, the epidemic had attained a fearful malignity towards the close of the month. The interments on the 27th are reported to have been about 150, of which 130 were Germans and Irish. This report is probably much exaggerated. We shall look with interest for an authentic account of the epidemic in the Western Lancet.

In Lexington, the whole number of deaths from cholera up to the 27th of June was 38, of which 17 were whites, and 21 blacks. The first fatal case occurred on the 11th of June, the day of the month on which the disease attained its height there in 1833. The number of victims to the epidemic in the Lunatic Asylum, at the last dates, was about 60. The first fatal case appeared in the Asylum on the 19th of May. Lexington, from present appearances, is likely to escape a severe visitation of the pestilence. But few cases were occurring about the end of the month.

In Louisville, as was mentioned in our last number, several well-marked cases of cholera appeared on the first of May, after a day of extraordinary heat, the thermometer having stood as high as 86° in the shade. From that time to the present, but few days have passed without a death from the disease in the city, and yet the number of fatal cases hardly now exceeds a hundred. Our colleague, Dr. Bell, a member of the Board of Health, has been at considerable pains to collect the statistics of cholera in the city, and we learn from an article written by him, in the Morning Courier, that after the disease had been prevalent fifty-five days, that is, up to the 24th of June, the number of persons who had died of it, was short of seventy. From the 25th to the 28th, inclusive, 30 deaths from cholera were reported by the Board of Health. The number of interments on the 27th was 22, the greatest number known in Louisville in one day in a period of twenty years. Of these, 9 were from cholera. The interments on the 28th were 19; of which 11 were from cholera. For many days, during which the epidemic attained this mortality, the thermometer, at sunrise, ranged from 72° to 76°, and rose by 3 o'clock, P. M. to 92°, in the shade. On the evening of the 27th, after a day of intense heat, a heavy shower of rain fell, and on the day succeeding there was a manifest improvement in the health of the city. The number of deaths reported on the 29th and 30th was 13.

As in Nashville, so here the complaint has manifested its greatest malignity in a few circumscribed localities. Dr. Bell, in the article referred to, states, that of the first seventy cases that occurred in the city, fifty-three were afforded by a space not exceeding four squares. On Fifth street, near the river, 28 deaths occurred; on Water street, between Fifth and Sixth, including the alley, 7; on Water street, and the alley between Third and Fourth, 11; on Water street, from Third to Second, 5; and in Strader's Row, on the river, 2. This is more than five-sixths of the mortality in the city up to the 25th, while the space in which it occurred is less than one-twentieth of the city limits. The region thus signalized by the epidemic is one of the most crowded in Louisville, and certainly the most filthy. Most of the victims in this locality were foreigners, principally Irish and Germans.

The epidemic has existed here in its mildest and most manageable form. The cases have, with remarkable uniformity, yielded to treatment when attended to at an early stage. Neglect or intemperance has been the cause of the fatal issue in all but a few exceptional instances.

